# Variation in flower size and shape of *Impatiens capensis* is correlated with urbanization in Montreal, Canada

**DOI:** 10.1002/ece3.10826

**Published:** 2023-12-13

**Authors:** Julie Faure, Valentine Volz, Simon Joly

**Affiliations:** ^1^ Institut de Recherche en Biologie Végétale Département de Sciences Biologiques Université de Montréal Montréal Quebec Canada; ^2^ Montreal Botanical Garden Montréal Quebec Canada

**Keywords:** flower shape and size, geometric morphometrics, pollinator visitation rates, urbanization

## Abstract

Urbanization is changing the conditions in which many species live, forcing them to adjust to these novel environments. Floral size and shape are critical traits for the reproduction of plants pollinated by animals as they are involved in the attraction of pollinators and in efficient pollination. Variation in size and shape could be affected by urbanization via its modification of the abiotic environment (habitat fragmentation, water availability, temperature, soil properties), or via its impact on the biotic environment of plants (pollination, herbivory). Although numerous studies have assessed the impact of urbanization on pollinator communities and many plant traits, few have investigated its impact on floral size and shape while quantifying the proportion of the total urbanization effect that is due to biotic interactions. In this study, we tested if urbanization and pollinator visitation rates affect the flower shape of the spotted jewelweed, *Impatiens capensis*. We quantified the size and shape of flowers in frontal and profile views using geometric morphometrics for 228 individuals from six populations from the region of Montreal, Canada. Pollinator visitation rates were estimated at each site and the main pollinators were found to be bumblebees, honeybees and hummingbirds. We found that floral size and shape are significantly correlated with urbanization as measured by the amount of vegetation in the surrounding environment of the plants (mean normalized vegetation index, NDVI) and by the visitation rates of bumblebees and honey bees. Partitioning of the total flower shape variation suggests that urbanization affects flower shape through abiotic factors and via its impact on pollinator visitation rates. While further studies from other cities are necessary to confirm the role of urbanization in shaping the floral shape of *I. capensis*, these results support the idea that urbanization could affect flower shapes.

## Graphical abstract

We found that the flower shape and size of the jewelweed is correlated with urbanization. Urban populations tend to have smaller flowers and sepals with shorter spurs. These changes are partly explained by vegetation cover and by variations in pollinator communities.

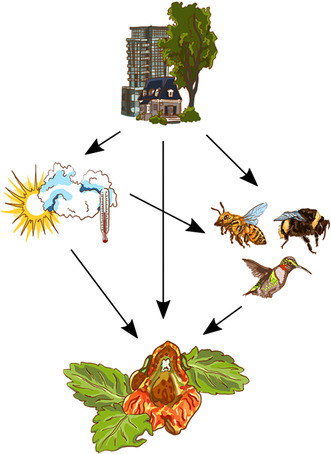

## 1 INTRODUCTION

Throughout the world, urbanization is reshaping the conditions in which many species live. Urbanization, the conversion process of land into more densified cities, impacts the environment in terms of pollution, temperature, habitat fragmentation and biodiversity. More than 55% of the world's population lives in cities, a proportion expected to rise to 68% by 2050 (United Nations, 2019). This augmentation and the fact that the area occupied by cities is increasing faster than the world's population (Liu et al., 2020) have a steady and increasing impact on the environment, forcing all forms of life to adjust if they want to persist in those habitats.

Communities of organisms present in cities are often very distinct from those of the surrounding natural environments. This is in part because cities impose environmental filters, such as habitat transformation, environment and human preferences, that select species with specific characteristics (Beninde et al., 2015; Williams et al., 2009). But cities also bring many introduced animals and plants that act as global homogenizers (McKinney, 2006). Yet, while the urban environment can modify the species composition, they also alter the abiotic environment and habitat to which species could adjust by phenotypic plasticity or natural selection (Alberti et al., 2017; Johnson & Munshi‐South, 2017).

Urbanization can affect organisms by modifying their abiotic environment such as pollution, habitat fragmentation, higher temperatures and soil properties (Santangelo et al., 2020). However, urbanization can also impact organisms by affecting their biotic environment, that is the species with which they interact (Irwin et al., 2020). Among trophic interactions, pollination mutualisms are particularly critical because of their importance to both animals and plants. While many animal pollinators rely on nectar and pollen as their main food source, approximately seven out of eight flowering plants rely on animals for their pollination (Ollerton et al., 2011). Therefore, changes in the presence, abundance, and timing of pollinators could affect plant communities, and vice‐versa, especially when phenological changes are not synchronized (Fisogni et al., 2020; Forrest, 2016).

Urbanization is known to impact pollinator communities and species (Geslin et al., 2013; Larson et al., 2014; Leong et al., 2016; Martins et al., 2017). This could be due to habitat fragmentation that limits the foraging potential, especially for small species (Greenleaf et al., 2007; Hung et al., 2017; Soga et al., 2014), to the modification of floral and nesting resources (Burdine & McCluney, 2019; Hamblin et al., 2018; Somme et al., 2016; Theodorou et al., 2017; Wray & Elle, 2015), and to changes in abiotic variables such as temperature (Hamblin et al., 2018) or soil composition (King & Buckney, 2000). Although previous studies have shown that it is impossible to define a single trend regarding the impact of urbanization on pollinator communities (Wenzel et al., 2020), the abundance, species richness, visitation rates of pollinators and pollen transfer generally decreases with higher levels of urbanization (Bates et al., 2011; Burdine & McCluney, 2019; Hamblin et al., 2018; Rivkin et al., 2020; Wenzel et al., 2020). Because pollinators generally impose strong selective pressures on floral traits (e.g. Galen, 1989; Gervasi & Schiestl, 2017; Sahli & Conner, 2011; Stanton et al., 1986), urbanization could indirectly impact plant fitness if changes in pollinator communities result in locally maladapted plant phenotypes (e.g. Gómez et al., 2009). Similarly, such modifications of pollinator communities could also lead to plant adaptation to match these new conditions.

**FIGURE 1:**
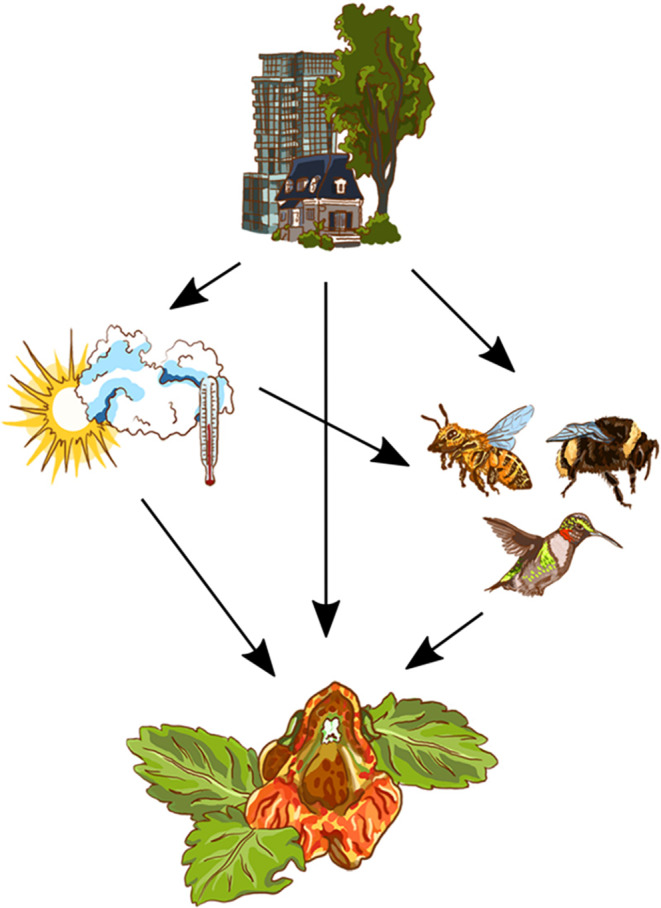
An illustration showing how urbanization could affect plant traits directly through the modification of the abiotic environment or via biotic variables (pollinators) that are themselves affected by the abiotic environment (credit: V. Volz).

Few studies have tried to tease apart the abiotic and biotic effects of urbanization on plants phenology and phenotype (Figure 1) (Neil et al., 2014; but see Rivkin et al., 2020; Ushimaru et al., 2014). Among the potential abiotic effects of urbanization on floral traits, population fragmentation could alter reproductive strategies that either favor long‐distance pollination or that allow better colonization or maintenance of small populations. For instance, a community‐level analysis of the Paris region has shown that wind‐pollinated plants tend to be positively correlated with increased urbanization (Kondratyeva et al., 2020). Higher temperatures present in cities and variations in nutrients could also affect floral phenology (Mimet et al., 2009). In contrast, a biotic effect via a decrease in pollinator abundance could result in an increase in floral display as a way to attract more pollinators if self‐fertilization or vegetative reproduction do not represent possible alternatives (Thomann et al., 2013). Finally, some studies underlined variations that could be due to urbanization via both abiotic and biotic effects. For instance, in *Commelina communis*, city populations had less stigma‐anther separation (herkogamy) and a higher ratio of hermaphrodite to male flowers (Ushimaru et al., 2014). These changes could be advantageous to colonize the small fragmented populations present in cities by increasing the probability of self‐fertilization, but it might also result from a change in pollinator communities that reduced pollination rates.

In this study, we wanted to test if urbanization could influence floral shape. The floral shape could be influenced directly by urbanization via the availability of resources that can affect flower size as well as shape via allometry effects (Strelin et al., 2021; Ushimaru & Nakata, 2001). But plant shape is also known to be influenced by the presence of pollinators because it is involved in both the attraction of pollinators and in the pollination efficiency by contributing to the mechanical fit between the pollinator and the reproductive organs of the flower (e.g. Galen, 1989; Muchhala, 2007). Yet, to date, there have been very few studies on the influence of urbanization on flower shape.

We investigated the impact of urbanization on the flower shape and size of *Impatiens capensis* in six populations from the region of Montreal, Canada. Previous studies have shown that the spur curvature of *I. capensis* has a strong genetic basis (Travers et al., 2003) and that the angle of the spur is correlated with fitness (Young, 2008). Moreover, a simulated pollinator decline showed selection for greater flower size in this species (Panique & Caruso, 2020). With its generalist pollination strategy (see Methods section for details), the documented role of flower size and shape in plant fitness, and the presence of pollen limitation in both urban and natural habitats (Barker & Sargent, 2020), *I. capensis* is a great candidate to study the impact of urbanization on flower size and shape.

The objectives of this study were to (i) test whether there is significant floral shape variation among populations at a city scale, if it is the case (ii) to test if urbanization explains the shape variation, and if so (iii) quantify what proportion of the effect is due modification of the pollinator communities. To do so, we quantified the shape of jewelweed flowers from 228 individuals from six populations in profile and frontal view using a semi‐3D geometric morphometric approach to test if urbanization and pollinator visitation rates could explain variation in floral shape.

## 2 METHODS

### 2.1 Study organism

**FIGURE 2:**
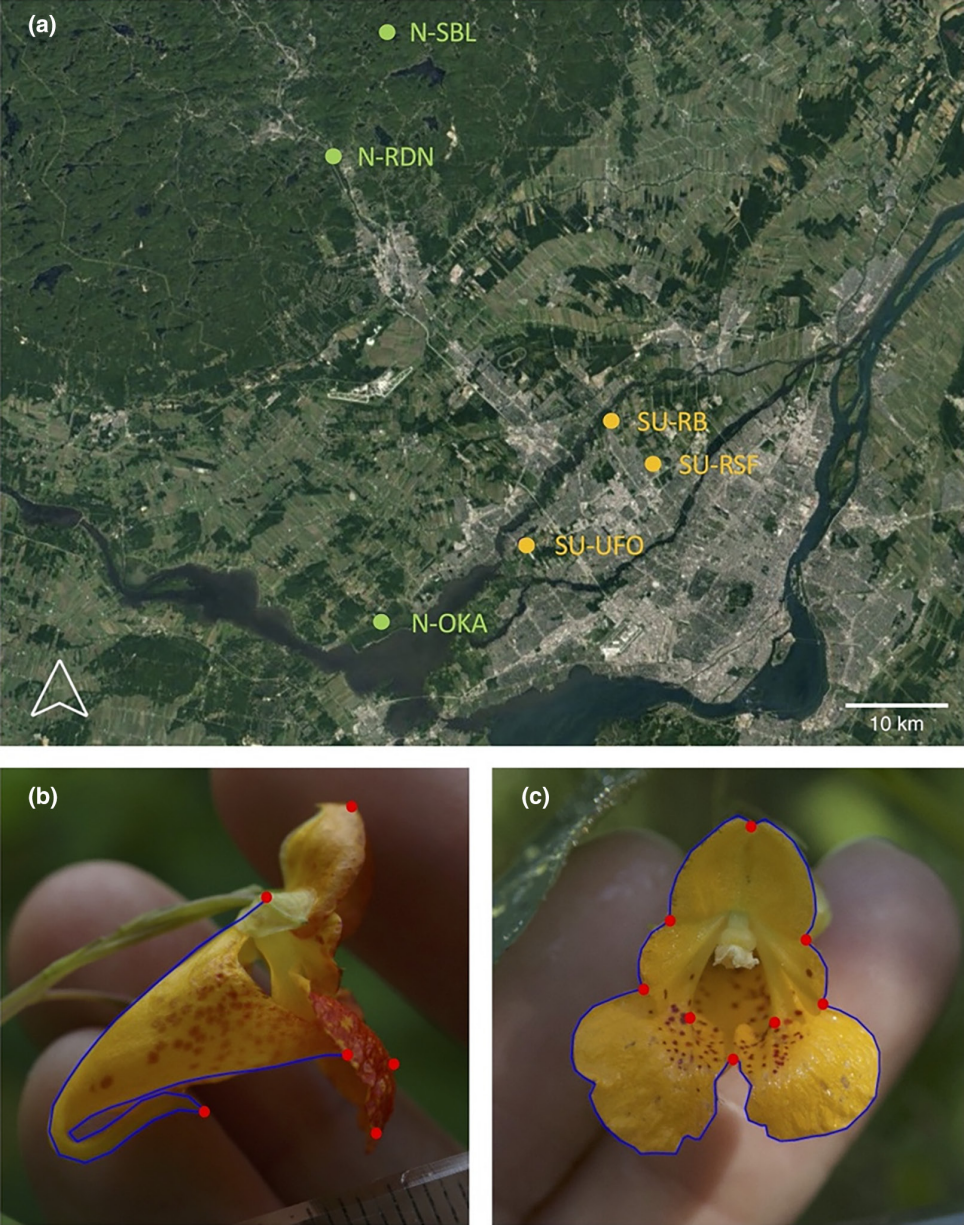
(a) Map showing the studied populations in the region of Montreal, Quebec, Canada (aggregate map of Landsat8 satellite images from the summers of 2017 and 2018 compiled with Google Earth Engine). Natural sites are in green, whereas suburban sites are in orange. Lower panels show the position of the landmarks (red dots) and semi‐landmarks (blue curves) on flower pictures in profile (b) and front (c) view (photo credits: J. Faure).

*Impatiens capensis* Meerk. is an annual species native to North America that grows in damp soils, often near open water, though it can also be found in urban areas (Barker & Sargent, 2020). The plant can reach two meters in height and it flowers between July and mid‐September. It has a mixed‐mating system with chasmogamous flowers that are pollinated by biotic vectors and cleistogamous flowers that allow autonomous self‐fertilization. Chasmogamous flowers are abundant in populations that receive a lot of direct sunlight, whereas cleistogamous flowers are present in all populations but represent the main type of flower in shaded populations (Waller, 1980). The chasmogamous flowers are protandrous (Rust, 1977), zygomorphic, orange‐yellow with red dots and they present a nectar spur on a highly modified sepal (Figure 2b). The fruit is a capsule with explosive dehiscence. *Impatiens capensis* has a generalist pollination strategy offering both pollen and nectar as a reward and is mainly pollinated by hymenopteran *Bombus* spp., *Apis mellifera*, *Archilochus colubris*, and sometimes *Vespula* spp. (Rust, 1977). It is also visited by nectar thieves that either collect nectar without touching the reproductive organs, make a hole in the spur to reach the nectar, or get nectar from a hole made previously (Rust, 1977).

### 2.2 Populations studied

Six populations of *Impatiens capensis* were studied. Three were located in suburban zones and three in more natural sites (Figure 2a). Because *I. capensis* generally lives in damp soils, it is rarely found in highly urbanized area. The suburban sites are located in the city of Laval (identified with the prefix “SU” for suburban). One population on Rang St‐François (SU‐RSF) is a prairie on the side of a calm road near a highway (N45.61418° W73.70214°). The Rue Bergeron population (SU‐RB) is at the extremity of a dead end and the population starts in a flowering prairie and continues by a stream in a forest (N45.652144° W73.752036°). The last suburban population is located near the golf course UFO (SU‐UFO) and is located along a path crossing a stream in a forest (N45.54913° W73.85662°). The three other populations are located in natural parks and ecological reserves (identified with the prefix “N”). One natural site is in the Oka National Park (N‐OKA), in a prairie along the road crossing the park (N45.48597° W74.02457°). Another natural population is in the park Rivière‐du‐Nord (N‐RDN) along a stream (N45.868984° W74.084186°). The last population is located along the road in a protected reserve near the Station de Biologie des Laurentides (N‐SBL) of the Université de Montréal, in the town of Saint‐Hippolyte (N45.974711° W74.020419°).

### 2.3 Quantification of urbanization

Urbanization was quantified at each site using the proportion of impervious surfaces and the presence of vegetation. The presence of vegetation was estimated from the Normalized Difference Vegetation Index (NDVI) index, a commonly used vegetation index (Esau et al., 2016; Himan et al., 2012; Pettorelli et al., 2011), whereas the proportion of impervious surfaces was estimated using the Global Man‐made Impervious Surface (GMIS) database (De Colstoun et al., 2017). A maximum NDVI [= (NIR‐Red)/(NIR + Red)] map was computed using Google Earth Engine with sentinel‐2 satellite data (Drusch et al., 2012) between 15 June 2020 and 31 August 2020, which coincide with the end of the blooming period of *I. capensis*. Tiles from the target areas were exported in .tif format, loaded in QGIS v 3.22.9 (QGIS Development Team, 2023) and merged. Water was removed using shapefiles of rivers and lakes (Canada open data).

We estimated the mean NDVI and the mean proportion of impervious surfaces for circles of 2 km radius around the populations to account for the potential foraging range of the pollinating insects. Although *Apis mellifera* is known to be able to forage up to 10 km from their hive (Beekman et al., 2004), most studies report foraging distances between 500 m and 2.5 km (Schneider & Hall, 1997; Visscher & Seeley, 1982; Waddington et al., 1994). *Bombus terrestris* is known to forage within 2.2 km from their nest (Kreyer et al., 2004; Osborne et al., 2008) and most smaller bees forage within 1 km from their nest (Greenleaf et al., 2007). A radius of 2 km thus seems adequate, although we note a very high correlation in mean NDVI (*r* = .983) and proportion of impervious surfaces (*r* = .917) between circles of 500 m and 2 km radius, suggesting that this decision is unlikely to affect the results. The mean NDVI and mean proportion of impervious surfaces were calculated in QGIS (QGIS Development Team, 2023) using the buffer and zone statistical tools. Water was removed from the estimates.

### 2.4 Pollinator observations

At each site, pollinators were observed on 2 non‐consecutive days, from August 8th to 30th 2019, during the blooming season of *I. capensis*. One day focused on morning pollination (8 a.m. to 2 p.m.) and the other on afternoon pollination (2 p.m. to 6 p.m.). Two people observed a given number of open flowers and noted all floral visitors. They noted if the visitors contacted the reproductive organs (pollinators), the identity of the visiting species, the time of the visit and the number of flowers visited by the animal. For three sites, a video camera was also used to record visits to additional flowers. Because it is difficult to identify insect species without capturing them, we classified the insects at the family level except for bumblebees (*Bombus* sp.) and honeybees (*Apis melifera*).

The abundance of each pollinator species was estimated on an hourly basis, that is the mean number of pollinators that were observed coming to the observed patch per hour, irrespective of the number of flowers it pollinates. Shannon and Simpson diversity indices were measured on these abundances per site, using the ‘diversity’ function from the vegan package (Oksanen et al., 2013). We also calculated the pollination rate per species, which is the number of visits of a pollinator species per flower, per hour. To illustrate the differences in pollinator visits among the populations, we performed a Correspondence Analysis on the pollinators' visitation rates per flower per hour.

### 2.5 Flower shape data and morphometry

One flower from 40 individuals per population (20 in Golf UFO due to its smaller size) was photographed in front and profile views (Figure 2b,c) with a ruler to account for size. Two pictures of each flower in the two positions were taken and processed independently to quantify the technical error involved in the photography and morphometric approaches. We noted the developmental stage of the flower, which was either male or female. The two phases are distinct because the stigma opening forces the stamens to fall by pushing the latter from below.

We used geometric morphometrics to quantify the flower shape. Profile photos were digitized using the TpsDig software (Rohlf, 2021). Six landmarks (homologous points) were used: one at the tip of the nectar spur, one at the point connecting the flower to the pedicel, one at the tip of the top petal, one at the top of the curve formed by the basal petal (semi‐homolog point), and one at the tip of the lower petals (Figure 2b). Two curves were used on the profile view: one follows the curve of the sepal from the point where it is attached to the pedicel to the tip of the spur, and another along the bottom part of the sepal from the tip of the spur to the end of the sepal. Each curve was quantified with 20 semi‐landmarks. Each photo was scaled with a ruler.

The frontal photographs were digitized with Fiji (Schindelin et al., 2012) using eight landmarks and six curves (Figure 2c). The landmarks were positioned at the tip of the standard (top) petal, at the intersection between the standard and wing petals, at the intersection between the wing and the standard petals, at the intersection between the two standard petals, and the two widest points of the flower tube opening. Two curves described the shape of the standard petal (eight semi‐landmarks each), two to quantify the shape of the side of the flower formed by the lateral petals (wings) (four semi‐landmarks each), and two curves of 14 semi‐landmarks each to describe the shape of the bottom petals.

Landmarks were imported in R (R Core Team, 2022) with the geomoprh package (Adams & Otárola‐Castillo, 2013). Flowers with photographs of poor quality or with large left–right asymmetry for the photos in front view were removed. We also removed two individuals that did not have spurs as this created problems with the morphometric analyses. We calculated the size of the flowers by using the area of the sepal that forms the pouch and the spur. This was done using the Polygon function of the sp R package (Bivand et al., 2013).

We performed a generalized Procrustes analysis that centers the digitized flowers, removes size and rotates them to superimpose all flowers for further analyses. The semi‐landmarks were superimposed by minimizing the Procrustes distance between the reference and the target flower shape in frontal photos. In profile view, the huge variation in spur orientation caused alignment problems if we allowed the semi‐landmarks to slide during the Procrustes analysis. We thus treated them as fixed landmarks. Following the generalized Procrustes analysis, outliers identified with the ‘plotOutliers’ function were discarded.

To analyze the frontal and profile shapes simultaneously and thus have a better idea of floral variation in 3D, the mean 2D coordinates of each individual, front and profile, were combined in a single 3D array, keeping the two sets of coordinates orthogonal (van de Kerke et al., 2020) to analyze both views simultaneously. To combine the datasets, a uniform *z* coordinate was added to all pictures in profile view (i.e. the original *x* coordinate of the point at the tip of the standard petal for pictures in frontal view); the *x* coordinates of the frontal pictures became *z* coordinates and the frontal pictures were attributed a uniform *x* coordinate (i.e. the *x* coordinate of the point connecting the flower to the pedicel in profile view).

### 2.6 Statistical analyses

Although the sites were categorized into suburban and natural categories, the variables quantifying urbanization varied within each category. We therefore decided to use the urbanization variables in the statistical test to quantify the effect of urbanization rather than the two categories.

The effect of urbanization on pollinators visitation rates was assessed by Canonical Correspondence Analysis (CCA) using the vegan R package. The best model was selected with the help of the step function in a forward fashion, selecting the model with the smallest AIC value.

We tested the effect of urbanization (NDVI and the proportion of impervious surfaces) and pollinator visitation rates on flower size using a linear model in R using the ‘lm’ function. All variables were included in the model, although we removed strongly multi‐colinear variables from the model (absolute Pearson correlation coefficient >0.9) to make sure that the variance inflation factor of the variables of the model was smaller than 2. To make sure the developmental stage did not interfere with the results, we then tested if interaction terms including the developmental stage were significant, in which case they were included in the model. All variables were tested after all others were included (type III tests).

To quantify the variation between independently digitized copies of the flowers, a Procrustes linear model (also called a Procrustes ANOVA or a PERMANOVA; Collyer et al., 2015) was performed on the profile and front shapes to quantify the variation between independently digitized copies of the flowers. A Procrustes linear model was also performed on the combined floral shape data to partition the variability in flower shape between individuals and populations. The models were fitted using the ‘procD.lm’ function of the package geomorph using 999 residual randomization and type III sum of squares. We tested for differences between populations in shape variance using homogeneity of multivariate dispersions (Anderson, 2006) with the ‘betadisper’ function of the vegan R package.

To quantify and test the effect of urbanization (NDVI and proportion of impervious surfaces) and the visitation rates of the pollinators on flower shape, we used a Procrustes linear model as described above. We first removed the variables that were strongly multicollinear (|*r*| > .9) and all other variables were included in the model. We then used a forward stepwise variable selection procedure to add interactions with size and developmental stage that could be significant to make sure that these variables did not affect our conclusions. We added interaction terms one by one, performing a comparison of models using an analysis of variance randomizing the null model residuals. At each step, the variable with the largest effect size that significantly improved the model was included to the model, until the model could not be improved. The final model was tested using type III sums of squares. To visualize the effect of each significant variable on flower shape, we visualized the effect of the vectors of regression coefficients on the mean flower shape and plotted the regression score of each variable with the same variable (see Drake & Klingenberg, 2008).

To tease apart the direct and indirect effects of urbanization on flower shape, we used variance partitioning (Peres‐Neto et al., 2006) to quantify the flower shape variation explained solely by urbanization, solely by pollinator visitation rates, and jointly by urbanization and pollinator visitation rates. Flower size was also added to the model because it was found to affect flower shape (see below). Variance partitioning was performed using redundancy analyses (RDA) with the vegan R package. The response matrix consisted of the significant principal components of a PCA of the shape dataset performed with the ‘prcomp’ R function. The principal components selected were those that explained more variation than expected at random according to a Brokenstick criteria. The pollinator data was the chi‐squared transformed rate visitation matrix (when more than one pollinator was included in the model) to avoid considering the shared absence of pollinators as evidence of similarity. The urbanization matrix consisted of the mean NDVI and the proportion of impervious surfaces. The partitioning of the total shape variation according to the independent matrices was done with the ‘varpart’ function of the vegan R package and the significance of the different fractions was tested by RDA and partial RDA.

## 3 RESULTS

### 3.1 Quantification of urbanization

We calculated the mean NDVI value and the percentage of impervious surfaces in a 2 km radius circle around each site (Table 1). The natural sites had a much higher mean NDVI value and three suburban sites had a greater proportion of impervious surfaces.

**TABLE 1:** Mean NDVI value and proportion of each type of habitat within a 500 m radius circle around each population.

Site	Mean NDVI	Percentage of impervious surfaces (%)
SU‐RSF	0.548	33.8
SU‐RB	0.615	28.2
SU‐UFO	0.600	31.4
N‐SBL	0.793	0
N‐OKA	0.775	14.4
N‐RDN	0.725	16.3

*Note*: Water was excluded from all estimates. Natural sites are identified with the prefix “N” and suburban sites are noted with the prefix “SU”.

### 3.2 Observed pollinators and visitors

A total of 107.5 h of observation were made, which corresponds to 3883 h of observation per flower when considering that several flowers were observed simultaneously. Insect visitors were classified into the following taxonomic groups: HYMENOPTERA: *Bombus* sp. (e.g. *Bombus impatiens*, *Bombus vagans*, *Bombus ternarius*), *Apis melifera*, *Xylocopa* sp., Halictidae (e.g. *Augochlorella aurata*), Megachilidae (e.g. *Anthidium florentinum*), Andrenidae (e.g. *Andrena* sp.), Sphecidae (e.g. *Sceliphron caementarium*), Vespidae (e.g. Vespula); DIPTERA: Syrphidae (*Syrphus* sp., *Hypocritanus fascipennis*, *Rhingia nasica*, *Toxomerus geminatus*); LEPIDOTERA: Papilionidae (e.g. *Papilio cresphontes*); and the ruby‐throated hummingbird (*Archilochus colubris*).

**FIGURE 3:**
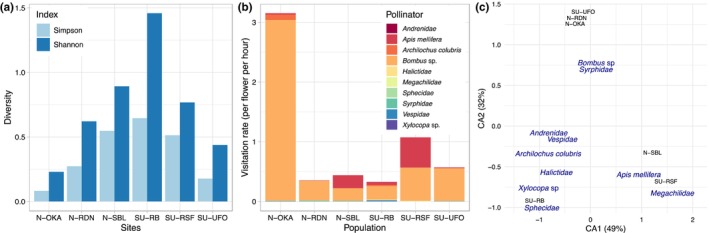
Pollinators diversity and visitation rates. (a) Simpson and Shannon pollinator diversity at each site based on abundance. (b) Pollinators visitation rates for each population. (c) Correspondence Analysis (CA) of the pollinators visitation rates. The CA plot is in scaling 2, which means that the angles between vectors are indicative of the correlation between them.

Species were considered pollinators when they made contact regularly with the reproductive organs. The most frequent pollinators were bumblebees (*Bombus* sp.) with a mean of 0.81 visit per flower per hour across populations, followed by honey bees (*Apis mellifera*; 0.13 visit per flower per hour) and to a lesser extent the ruby‐throated hummingbird (*Archilochus colubris*; 0.055 visit per flower per hour; Figure 3b). All other pollinators were rarely observed and unlikely to play a major role in the pollination of *I. capensis* (visitation rates <0.007 visit per flower per hour) and were not considered further for the study of flower shape. Very few nectar thieves were observed and these were Vespidae (three cases observed) and Megachilidae (two cases observed); these were not considered further due to the small sample sizes.

The flower populations with the highest visitation rates were not necessarily the ones with the greatest diversity. The populations SU‐RB, N‐SBL and SU‐RSF had the highest diversity, whereas N‐OKA, SU‐RSF and SU‐UFO were found to have the highest visitation rates (Figure 3a,b). The Correspondance Analysis (CA) of the pollinator visitation rates shows that bumblebees and Syrphidae are more associated with the populations N‐OKA, SU‐UFO and N‐RDN, that Megachilidae and *Apis mellifera* are more associated with the populations SU‐RSF and N‐SBL, and that SU‐RB has more minor pollinators such as Halictidae, Xylocopa and Sphecidae (Figure 3c).

We evaluated the impact of urbanization on the pollinator visitation rates using Correspondence Canonical Analysis (CCA). The stepwise selection procedure chose a model with impervious surfaces and mean NDVI had the lowest AIC score. This model explained 64.3% of the variation in pollinator communities and was statistically significant (CCA: _adj_ *R* ^2^ = .643, *p* = .04028).

### 3.3 Variation of floral shape

A total of 14 photos in front view were discarded because of a poor quality or strong asymmetry and a further 25 were removed because they were identified as outliers. This left 192 individuals in the analysis that had both front and profile data. Following the general Procrustes analysis, a Procrustes linear model was used to quantify the variation between the two digitized copies of each picture. The variation was 3.2% for the photos in profile views and 15.4% for the photos in face view. We computed the mean shape between the two pictures that were digitized for each specimen and then created virtual 3D models for each individual. These were used for the following analyses.

The Procrustes linear model showed significant differences in shape between the different populations (*R* ^2^ = .095; *p* < .001). In contrast, we did not find evidence that shape variation differed between populations (Homogeneity of multivariate dispersions; *R* ^2^ = .034; *p* = .258).

### 3.4 Effect of pollinators and urbanization on floral size and shape

The mean NDVI and the proportion of impervious surfaces were strongly correlated (*r* = −.949) as were the visitation rates of *Bombus* sp. and *Archilochus colubris* (*r* = .946). We thus excluded the proportion of impervious surfaces and the visitation rates of *Archilochus colubris* from the models on size and shape. The variance inflation factors were <2 after these variables were removed.

**FIGURE 4:**
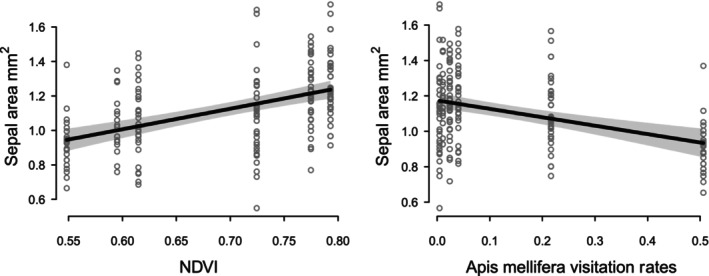
Plots showing the relationship between flower size and the mean NDVI and *Apis mellifera* visitation rates while holding the other variable of the model constant (see Table 2). The gray area indicates the confidence interval around the slope.

For the multiple regression on flower size, as estimated by the area of the pouched sepal, no interaction terms including the developmental stage were significant. The final model explained 35% of the total variation (_adj_ *R* ^2^ = .35, df = 183, *p* < .001) and indicated that flower size is significantly affected by mean NDVI and the visitation rates of *Apis mellifera* (Table 2, Figure 4). An increase of 0.2 value of mean NDVI in a 2 km radius circle around the population resulted in an increase of 0.238 mm^2^ in the area of the pouch sepal (*p* < .001; Table 2), indicating that flowers are smaller in more urban areas that have lower NDVI mean values (Table 1). Also, a decrease of 0.2 in *A. mellifera* visitation rates was associated with a decrease of 0.095 mm^2^ in the area of the pouch sepal (*p* < .001; Table 2).

**TABLE 2:** Linear model of the flower size on urbanization.

Variable	Value	SE	Sum sq	df	*F* value	*p*‐value
Intercept	0.3022	0.0184	4.259	1	5.540	.0197
Male flowers	0.0139	0.0364	0.0057	1	0.1458	.7030
NDVI	1.191	0.1772	1.759	1	45.13	<.001
*Bombus* sp.	−0.0137	0.0148	0.0335	1	0.8594	.3551
*Apis mellifera*	−0.4752	0.0942	0.9917	1	25.44	<.001
Residuals			7.134	183		

*Note*: Significance was tested with type III sums of squares.

**FIGURE 5:**
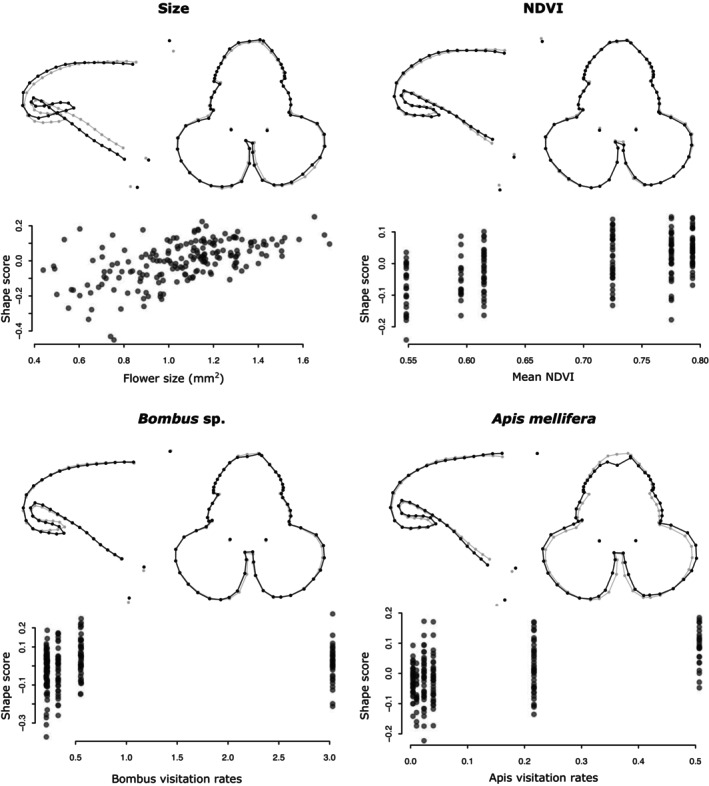
Effect of the significant variables of the Procrustes regression on flower shapes (Table 3). The top panels show the mean flower shape across populations in gray and the effect of an increase of half the range in each variable in black as predicted by the Procrustes linear model, that is an increase of 0.64 mm^2^ in flower size, an increase of 0.12 of NDVI, an increase of 1.41 visits per flowers per hour for *Bombus* sp. and an increase of 0.25 visits per flower per hour for *Apis mellifera*. The bottom panels show the flower shape scores for the flowers included in the study on the *y*‐axes according to each variable in the dataset on the *x*‐axes.

The best Procrustes linear model according to the model selection included an interaction between size and NDVI in addition to the individual variables (Table 3). All individual variables were found to significantly explain the flower shape. The size accounted for 3.7% of the total shape variation (*p* < .001; Figure 5) and the fitted model suggests that an increase in flower size resulted in a thicker pouch and a more curved spur (Figure 5). In contrast, no clear effect was observable on the front shape (Figure 5). The mean NDVI accounted for 1.5% of the total shape variation (*p* = .003; Table 3) and the model suggests that an increase in mean NDVI results in a slightly narrower rear pouch and a longer spur (Figure 5). As with size, little effect was observable on the front flower. The *Bombus* sp. and *A. mellifera* visitation rates explained 1.1% and 2.5% of the shape variation, respectively, and increases in visitation rates is associated with straighter spurs for *Bombus* sp. and a slightly longer spur for *A. mellifera* (Figure 5).

**TABLE 3:** Procrustes linear model.

Variable	df	*R* ^2^	*F*	Effect size (*Z*)	*p*‐values
Size	1	.037	8.29	4.83	.001
Developmental stage	1	.020	2.23	2.41	.012
NDVI	1	.015	3.44	2.75	.003
Bombus	1	.011	2.38	2.06	.018
Apis	1	.025	5.52	3.52	.001
Stage:NDVI	1	.023	2.56	2.77	.005
Residuals	183	.818			

*Note*: Significance based on 999 randomization of the residuals using a type III sums of squares.

### 3.5 Partitioning of variation

The Principal Component Analysis (PCA) of the flower shape resulted in 10 principal components that explained a significant proportion of variance (84% in total). In addition to the NDVI and *Archilochus colubris* visitation rates that were dropped in the previous analyses, we had to further drop the *Bombus* sp. visitation rates to remove collinearity in the analysis of variation partitioning.

**FIGURE 6:**
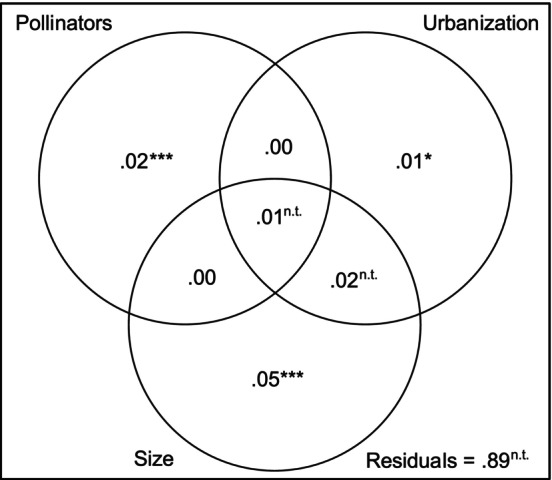
Venn diagram illustrating how the variation in floral shape is partitioned among size, pollinators and urbanization components according to a RDA analysis of the first 10 shape principal components. The values represent the adjusted *R* ^2^ values and values smaller than zero were set to zero. The residuals represent the unexplained variance in the model. *p < .05; ***p < .001, n.t. = not testable.

The urbanization, pollinator visitation rates and the flower size explained 11.3% of the floral shape variation (RDA: _adj_ *R* ^2^ = .113, *n* = 191, *p* < .001; Figure 6). The common fraction explained by flower size, the pollinator visitation rate and the urbanization represents 1% of the total shape variation, and the fraction explained by urbanization and size but excluding pollinator visitation rates represents 2% of the total variation (Figure 6). Size alone (excluding the effects of urbanization and pollinator visitation rates) explained 5% of the variation in floral shape (partial RDA, *p* < .001), urbanization alone explained 1% (partial RDA, *p* = .031), and pollinator visitation rates 2% (partial RDA, *p* < .001; Figure 6).

## 4 DISCUSSION

In this study, we used geometric morphometrics to test the hypothesis that urbanization affects the floral shape of *Impatiens capensis*. The first step in order to reach this objective was to investigate whether there was a significant floral shape variation between populations at the geographical scale of a city. Six populations were selected that represent a gradient in urbanization intensity. Urbanization correlates positively with a greater proportion of impervious surfaces and lower vegetation coverage in a circle of 2 km radius around populations (Table 1). We found a significant variation in shape between populations, with 9.5% of the total variation observed among populations.

We next tested whether flower shape and size were correlated with the degree of urbanization of the populations and found that both the size and shape were significantly correlated with urbanization. The shape was also affected by the size and the developmental stage of the flower. More specifically, among our populations, an increase in urbanization via a reduction in vegetation around the populations correlates with smaller flowers (Figure 4) and a shorter spur (Figure 5). And because the flower size also has an effect on shape, the smaller flowers of the suburban populations imply that the spurs are less curved even though this is not a direct effect of urbanization.

Pollinator visitation rates were also found to correlate with flower size and shape. Higher visitation rates of *Apis mellifera* are associated with smaller flowers and a slightly longer spur, whereas higher visitation rates of *Bombus* sp. are associated with straighter spurs.

Because urbanization could impact flower shape via abiotic variables or through its impact on pollinator communities (Figure 1), we used variation partitioning to quantify these effects. Because not all the variation in shape was included in this analysis (we used the significant principal components), the exact percentage of variation should be interpreted with caution. Nevertheless, the variance partitioning suggests that urbanization affects floral shape through abiotic factors (1% of the explained variation) but also potentially via a modification of the pollinator communities (1%; Figure 6). Because this fraction is also coexplained by size, it is possible that the effect of urbanization via the pollinators on shape is a side effect of selection by pollinators on flower size via allometry effects.

Together, our results suggest a correlation between flower shape and urbanization through both abiotic and biotic variables. Urbanization could affect flower size by affecting the availability of resources such as water or nutrients (Kuppler et al., 2021; Kuppler & Kotowska, 2021; Lambrecht & Dawson, 2007). Our results suggest that urbanization might also affect flower shape for the same reason via allometric effects. But our results also show that there is a significant amount of variation explained solely by urbanization, independent of size and pollinator visitation rates, hinting for a role of abiotic variables in shaping the Impatiens flowers. We also found that urbanization might affect flower shape via its impact on pollinator communities and that this shape effect is correlated with variation in flower size. This could occur, for instance, if urbanization was correlated with increased *Apis mellifera* pollination rates, which itself correlates with smaller flower sizes that could also affect flower shape via allometric effects. The correlation of pollinator visitation rates with flower shape, both separately and via its influence by urbanization, is not a surprising result as pollinator communities are well known to drive flower shape evolution (Galen, 1989; Gervasi & Schiestl, 2017; Sahli & Conner, 2011; Stanton et al., 1986). However, the fact that urbanization and pollinator visitation rates jointly explained floral shape variation supports our hypothesis that urbanization impacts flower shape through both abiotic and biotic effects.

We acknowledge that several factors prevent us from reaching firm conclusions regarding the impact of urbanization on floral size and shape. First of all, we sampled populations from a single city and the urbanization gradient is spatially structured, which means that it is possible that factors other than urbanization affected flower size and shape in our data. It will thus be very important to repeat this analysis in more cities to confirm the trends observed here. The addition of more populations would also allow to better identify the exact role of each variable in the system by increasing the statistical power. Moreover, our analyses assume that the flower shape and the pollinator visitation rates measured are typical for each population. Sampling flower shape and pollinator visitation rates over several years would certainly be interesting to quantify plasticity in flower shape and annual variation in pollinator visitation rates. Nevertheless, the results demonstrate that *I. capensis* is an interesting model to study the influence of urbanization on flower shape and that the geometric morphometric approach we used has the potential to provide information on this question.

It is interesting to observe an effect of size on flower shape, especially spur curvature. The lack of a significant interaction between size and NDVI in the model also suggests that this effect is found in all populations studied. Spur curvature is a well‐studied character in *I. capensis* and is known to be under strong genetic control (Travers et al., 2003). However, the allometric relationship found here highlights the importance of taking flower size into account when studying spur curvature.

We used a virtual 3D geometric morphometric approach (van de Kerke et al., 2020) to analyze simultaneously the front and profile shapes of the flowers. There was much more variation involved in the positioning of the landmarks for the front shapes (15.4%) compared to the profile shapes (3.2%), suggesting that it is more difficult to quantify the front shapes. But our results also suggest that size and urbanization do not strongly correlate with variation in the front shapes of flowers (Figure 5).

*Impatiens capensis* is a pollination generalist that can be pollinated by several insect pollinators as well as hummingbirds. This increases its chances to survive changes in its biotic niche such as the loss of one pollinator in a given population. Pollination generalists have often been associated with habitats characterized by poor pollinator density and diversity (Armbruster & Baldwin, 1998; Barrett, 1996; Waser et al., 1996), and as such this should also be a successful strategy to cope with environmental changes such as the spread of urbanization.

We found that urbanization may affect the evolution of the size and shape of flowers of *I. capensis*, and that this effect likely occurs both through abiotic effect and via its impact on the composition on the pollinator communities. While our results remains to be confirmed by the study of more populations from other cities, they suggest that in addition to being able to be pollinated by a range of pollinators, *I. capensis* may adapt to urban environments by rapidly evolving to novel pollinator communities via modifications in its floral size and shape.

## AUTHOR CONTRIBUTIONS

**Simon Joly:** Conceptualization (equal); formal analysis (supporting); methodology (equal); writing – review and editing (lead). **Valentine Volz:** Formal analysis (supporting); investigation (supporting); writing – review and editing (supporting). **Julie Faure:** Conceptualization (equal); formal analysis (lead); investigation (lead); methodology (equal); writing – original draft (lead); writing – review and editing (supporting).

## CONFLICT OF INTEREST STATEMENT

The authors declare no conflicts of interest.

### OPEN RESEARCH BADGES

This article has earned an Open Data badge for making publicly available the digitally‐shareable data necessary to reproduce the reported results. The data is available at https://doi.org/10.5061/dryad.63xsj3v77.

## Data Availability

The data that support the findings of this study are openly available in Dryad at https://doi.org/10.5061/dryad.63xsj3v77. Here is a temporary link to access the unpublished dataset during the reviewing process: https://datadryad.org/stash/share/ogDEbio_5oq3QPjVo1X_f0rgCzM5pw79H4HP8ip7XpI.
